# Role of Acetaldehyde and Dysregulated Mitophagic Lysosomal Processing in Chronic-Binge Ethanol-Induced Liver Injury

**DOI:** 10.3390/ijms262311608

**Published:** 2025-11-29

**Authors:** Devadoss J. Samuvel, Emory Foerster, Li Li, Amir K. Richardson, Patrick M. Wooster, John J. Lemasters, Zhi Zhong

**Affiliations:** 1 Department of Drug Discovery & Biomedical Sciences, Medical University of South Carolina, Charleston, SC 29425, USAlemaste@musc.edu (J.J.L.); 2 Department of Biochemistry & Molecular Biology, Medical University of South Carolina, Charleston, SC 29425, USA

**Keywords:** acetaldehyde, alcohol-associated liver disease, Alda-1, lysosome, mitochondrial biogenesis, mitochondrial damage-associated molecular patterns, transcription factor-EB

## Abstract

Chronic binge drinking is common among patients with alcohol-associated steatohepatitis. Therefore, we tested the hypothesis that chronic binge ethanol exposure disrupts mitophagic processing and stimulates release of mitochondrial damage-associated molecular patterns (mtDAMPs), thereby promoting hepatic inflammation and fibrosis after chronic binge ethanol (CBE) exposure in mice using the National Institute of Alcohol Abuse and Alcoholism model. After CBE, hepatic steatosis, liver injury, inflammation, and hepatic stellate cell (HSC) activation occurred. Alda-1, an aldehyde dehydrogenase-2 activator, attenuated these changes. After CBE, mitochondrial depolarization (mtDepo) occurred in ~85% hepatocytes, and mitophagy-associated proteins increased, which Alda-1 blunted. By contrast, transcription factor-EB (master regulator of lysosomal biogenesis) and lysosomal markers decreased, indicating disrupted lysosomal processing. After mitophagy, mitochondrial biogenesis (MB) restores mitochondrial mass and function. After CBE, peroxisome proliferator-activated receptor gamma coactivator-1 alpha (MB regulator), mitochondrial transcription factor-A, oxidative phosphorylation proteins, and fatty acid oxidation all decreased, which Alda-1 largely restored. After CBE, serum mtDAMPs (mitochondrial DNA and cytochrome c) increased 3- to 10-fold. In vitro, mitochondrial DNA stimulated macrophage and HSC activation, which was prevented by toll-like receptor-9 inhibition. In conclusion, CBE increases mtDepo in an acetaldehyde-dependent fashion, leading to mitophagic overburden, disruption of mitochondrial homeostasis, mtDAMP release, and ultimately development of liver inflammation and injury.

## 1. Introduction

Long-term excessive ethanol (EtOH) consumption leads to alcohol-associated liver disease (ALD), which comprises steatosis, hepatitis, and fibrosis/cirrhosis and culminates in end-stage liver disease and/or liver cancer [1,2]. Patients with severe alcohol-related hepatitis have a poor prognosis with a short-term mortality of 20–40% within 3 months of diagnosis [3,4,5]. In the US during 2022, 46% of liver disease deaths among people ≥ 12 years old were due to ALD [3]. Among all cirrhosis-induced deaths, ~50% were alcohol-related [3]. In 2016, ALD replaced hepatitis C virus infection to become the leading cause of liver transplantation [3]. Moreover, since the COVID-19 pandemic, the prevalence of ALD has rapidly increased [6], and this increase was sustained after the pandemic [7].

In alcohol-addicted patients, drinking cessation is difficult to achieve. Therefore, investigations on the mechanisms underlying the initiation and progression of ALD continue to be of high importance for identification of new therapeutic targets. ALD is a multistage disease with a pathogenesis mediated by multiple-hit mechanisms [8,9]. Early manifestations of hepatocyte injury by EtOH are abnormalities of mitochondria, such as megamitochondria and mitochondrial DNA (mtDNA) deletions [10,11]. EtOH selectively decreases mitochondrial glutathione, causes mitochondrial oxidative stress, and alters fatty acid β-oxidation and oxidative phosphorylation (OXPHOS) in mitochondria [11,12,13,14,15]. However, steps linking mitochondrial dysfunction to ALD are not well elucidated.

**Scheme 1:**
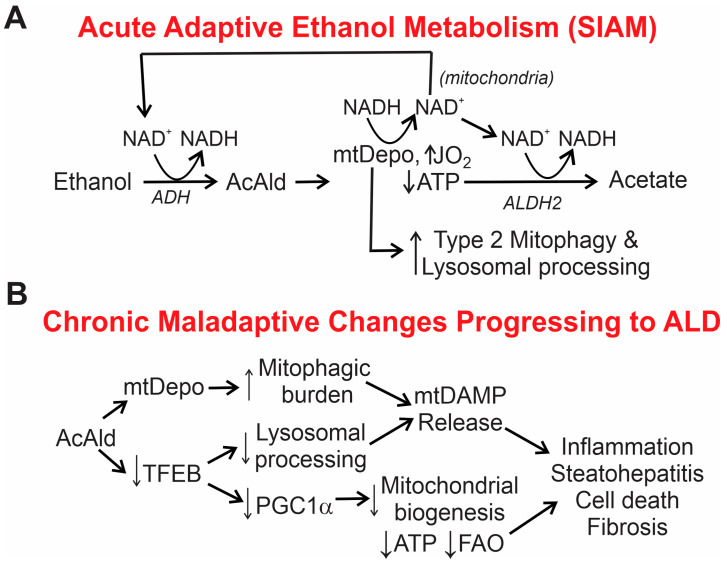
Dysregulated mitophagic processing as a tipping point that mediates the transition from adaptive to maladaptive responses leading to hepatitis and fibrosis in alcohol-associated liver disease. (**A**) Ethanol (EtOH) undergoes two-step oxidation to acetaldehyde (AcAld) and then to acetate, a process occurring predominantly in the liver. After acute EtOH consumption, mitochondrial depolarization (mtDepo), uncoupling, and increased oxygen consumption (↑ JO2) occur. These alterations enhance regeneration of nicotinamide adenine dinucleotide (NAD^+^), thus promoting more rapid detoxifying oxidation of EtOH by alcohol dehydrogenase (ADH) and AcAld by aldehyde dhydrogenase-2 (ALDH2). mtDepo in turn activates Type 2 mitophagy, which removes damaged mitochondria, a protective mechanism. Mitochondrial remodeling and biogenesis occur subsequently to restore mitochondrial homeostasis. (**B**) After chronic EtOH exposure, mtDepo continues to increase mitophagic burden, whereas prolonged exposure to EtOH and AcAld decreases transcription factor-EB (TFEB) expression and lysosomal processing of mitophagosomes, leading to release of proinflammatory and profibrotic mitochondrial damage-associated molecular patterns (mtDAMPs). Decreased TFEB may also lead to decreases of peroxisome proliferator-activated receptor gamma coactivator-1 alpha (PGC1α), thus suppressing restoration of mitochondrial homeostasis and function, such as ATP production and fatty acid oxidation (FAO). These alterations act as a tipping point from adaptation to maladaptation, leading to downstream pathogenic consequences of ALD, such as inflammation and fibrosis.

The liver is the principal organ that metabolizes EtOH and its toxic metabolite, acetaldehyde (AcAld), thus protecting other organs (Scheme 1A). After EtOH consumption, EtOH metabolism and mitochondrial respiration rapidly increase, a phenomenon named Swift Increase of Alcohol Metabolism (SIAM), which occurs both in human and animals [16,17]. In theory, increased respiration should promote ATP production and fatty acid oxidation (FAO), but ATP production actually decreases, and steatosis occurs after EtOH exposure [18,19,20]. Therefore, mitochondrial uncoupling and depolarization may stimulate respiration during SIAM (Scheme 1A). Using intravital multiphoton microscopy, a technique that allows direct visualization of hepatic mitochondrial polarization status in living animals, we observed dose-dependent mitochondrial depolarization (mtDepo) and decreased NAD(P)H autofluorescence in vivo after acute EtOH treatment [20]. Moreover, acute EtOH paradoxically decreased hepatic ATP by ~60% [20]. Together, these data indicate that mitochondrial uncoupling indeed occurs after acute EtOH in vivo. Such mitochondrial uncoupling after EtOH promotes more rapid oxidation of EtOH and AcAld by increasing NAD^+^ supply for alcohol dehydrogenase (ADH)- and aldehyde dehydrogenase-2 (ALDH2)-dependent EtOH metabolism (Scheme 1A), which is an adaptive process.

Inhibition of ADH and to a lesser extent of EtOH-oxidizing cytochrome P450 2E1 blunts SIAM [21,22,23]. Deficiency of the AcAld-generating enzyme ADH decreases mtDepo after acute EtOH by ~70%, and cytochrome P450 2E1 (CYP2E1) deficiency and cytochrome P450 inhibition decrease mtDepo by ~20%, consistent with the greater role of ADH in overall EtOH oxidation [20]. Also, in support of AcAld as the driver of mtDepo, ALDH2 activation by Alda-1 to eliminate AcAld more rapidly decreases mtDepo after acute EtOH, whereas ALDH inhibition with disulfiram enhances mtDepo [20]. Thus, increased AcAld from EtOH metabolism promotes mtDepo after EtOH treatment. Overall, intrahepatic AcAld formation after EtOH triggers mitochondrial adaptations, namely SIAM and mtDepo, to accelerate detoxifying EtOH and AcAld oxidation.

The occurrence of mtDepo after acute EtOH drives Type 2 (damage-associated) mitophagy in the liver, which is followed by increased lysosomal processing of mitophagosomes to remove damaged mitochondria after acute EtOH treatment [24] (Scheme 1A). Within 24 h following acute EtOH treatment by gavage, mtDepo reverses in over 85% of hepatocytes, suggesting recovery of mitochondrial homeostasis and function [20]. Under such circumstances, liver injury is very mild and no inflammation occurs [20].

Nonetheless, prolonged exposure to excessive EtOH causes ALD manifested by steatohepatitis and liver fibrosis/cirrhosis. How mitochondrial alterations during chronic EtOH exposure contribute to development of ALD remain incompletely understood. Chronic binge drinking is a typical drinking pattern for humans with alcohol use disorder and is often found in patients with alcohol-associated steatohepatitis [25,26]. Binge drinking superimposed on chronic drinking exacerbates and accelerates development of ALD [25,27]. Therefore, the objective of this study was to elucidate the relationship among mtDepo, mitophagy, lysosomal processing, and subsequent liver injury, inflammation, and fibrosis after chronic binge EtOH (CBE) exposure. We hypothesized that after CBE the adaptive response to EtOH causes recurrent or sustained mtDepo, leading to overburdened and dysfunctional mitophagic processing and resulting in disruption of mitochondrial homeostasis with release of proinflammatory and profibrotic mitochondrial damage-associated molecular patterns (mtDAMPs) [28,29]. As a result, the initial adaptive responses become maladaptive events, leading to progression of ALD.

## 2. Results

### 2.1. Chronic Binge Ethanol Increases Malondialdehyde-Acetaldehyde Adducts in the Liver: Blunting by Alda-1

**Figure 1:**
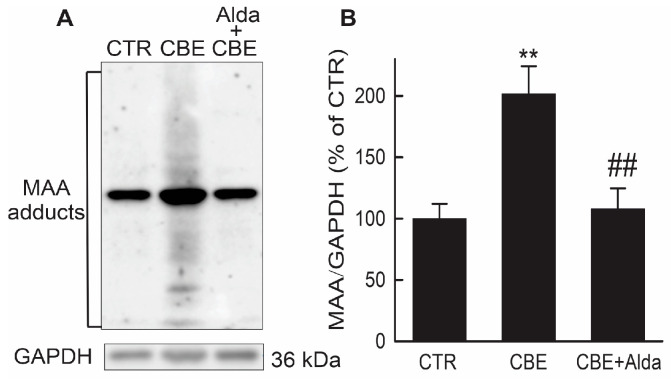
Increase in hepatic malondialdehyde-acetaldehyde adducts after chronic binge ethanol treatment: prevention by Alda-1. Female C57Bl/6 mice were fed control (CTR) or EtOH liquid diet and gavaged with one dose of sucrose dextrin or EtOH (5 g/kg) on day 16 (CTR and CBE). Mice also received Alda-1 (Alda) or vehicle during CBE treatment. Livers were harvested 9 h after gavage for detection of MAA adducts and house-keeping protein glyceraldehyde-3-phosphate dehydrogenase (GAPDH) by immunoblotting. (**A**) Representative immunoblot images; (**B**) densitometric quantification of immunoblot images. ** *p* < 0.01 vs. CTR. ## *p* < 0.01 vs. CBE. Values are means ± SEM (n = 4/per group).

Oxidation of EtOH by ADH and CYP2E1 produces AcAld, which is degraded by mitochondrial ALDH2 to acetate. AcAld reacts rapidly with other aldehydes/proteins to form adducts that are potentially toxic to mitochondria and may contribute to EtOH hepatotoxicity [30]. Malondialdehyde-acetaldehyde adducts (MAA) are hybrid AcAld adducts that are used as a surrogate for AcAld production [31,32]. Hepatic MAA adducts in female mice increased 102% after CBE treatment. With Alda-1 treatment that activates ALDH2 to accelerate AcAld degradation, MAA only increased 8%, which was not statistically different from the control mice (CTR) (Figure 1).

### 2.2. Chronic Binge Ethanol Causes Hepatic Steatosis, Injury, Inflammation, and Stellate Cell Activation: Attenuation by Alda-1

**Figure 2:**
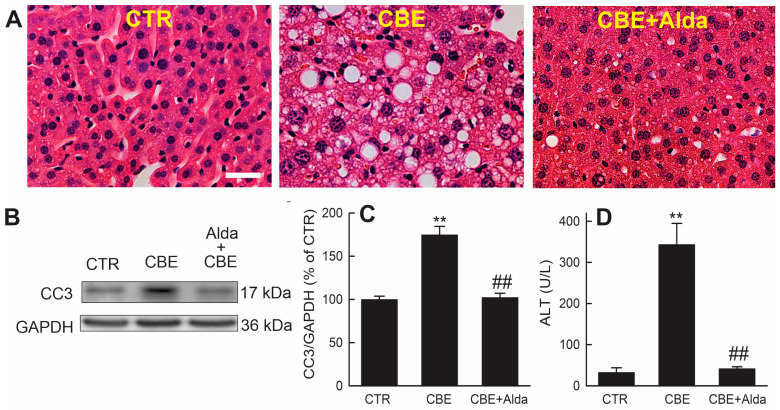
Liver steatosis, injury, and leukocyte infiltration after chronic binge ethanol treatment: suppression by Alda-1. Conditions are as in Figure 1. (**A**) Representative images of H&E-stained liver sections. Bar is 20 µm. (**B**) Representative immunoblot images of cleaved caspase-3 (CC3) and housekeeping protein GAPDH. (**C**) Densitometric quantification of CC3 immunoblot images. (**D**) Serum ALT. ** *p* < 0.01 vs. CTR. ## *p* < 0.01 vs. CBE. Values are means ± SEM (n = 4/per group).

Images of H&E-stained liver sections of female mice are shown in Figure 2A. No pathological alterations were observed in livers of mice fed CTR diet. After CBE treatment in female mice, moderate steatosis occurred. Fat droplets were primarily microvesicular, but macrovesicular fat droplets were present in some hepatocytes (Figure 2A). Scattered necrosis of hepatocytes and leukocyte infiltration also occurred (Figure 2A). Alda-1 treatment attenuated these pathological alterations (Figure 2A). Cleaved caspase-3 (CC3), an indicator of apoptosis, increased 75% after CBE treatment (Figure 2B,C). Alda-1 blocked apoptosis after CBE. Serum alanine aminotransferase (ALT), an indicator of liver injury, increased from a basal level of 33 U/L to 344 U/L after CBE treatment. Alda-1 decreased serum ALT to 42 U/L (Figure 2D). In male mice, similar hepatic pathological changes occurred after CBE treatment, which was again diminished by Alda-1 (Supplementary Materials, Figure S1). Alda-1 also decreased ALT from 396 U/L to 54 U/L in male mice after CBE treatment (Figure S2).

**Figure 3:**
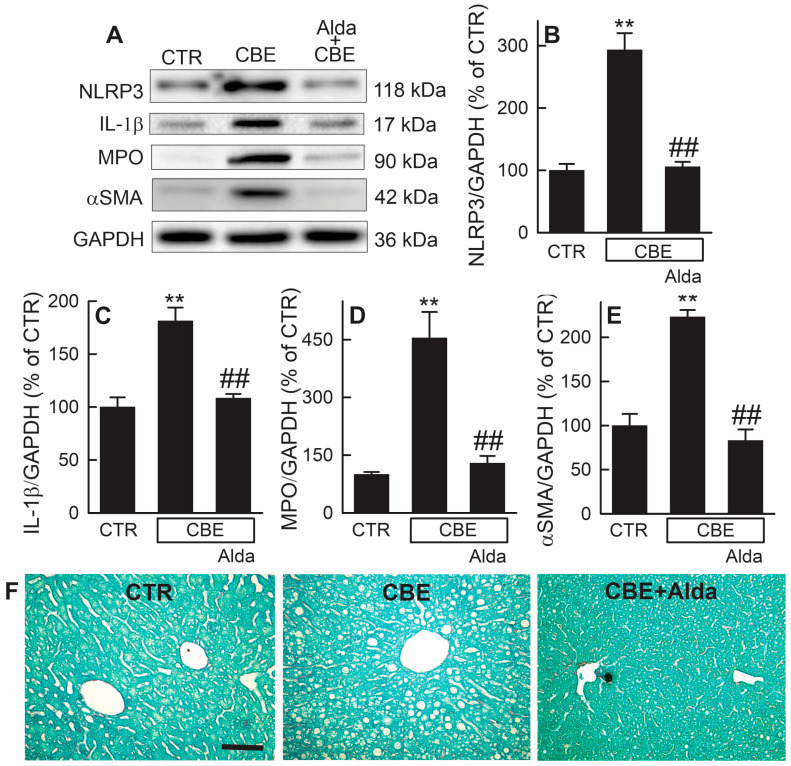
Hepatic inflammasome activation, polymorphonuclear cell infiltration and stellate cell activation after chronic binge ethanol treatment: prevention by Alda-1. Conditions are as in Figure 1. (**A**) Representative immunoblot images of NLRP3, IL-1β, MPO, and housekeeping protein GAPDH. (**B**–**E**) Densitometric quantification of immunoblot images. (**F**) Representative images of Sirius red/Fast green-stained liver sections. ** *p* < 0.01 vs. CTR. ## *p* < 0.01 vs. CBE. Values are means ± SEM (n = 4/per group). (**F**) Representative images of Sirius red/Fast green-stained liver sections. Bar is 50 µm.

Inflammasome activation is a key mediator of inflammatory responses, leading to maturation of proinflammatory cytokines interleukin-1β (IL-1β) and IL-18 [33]. NOD-like receptor protein 3 (NLRP3) increased 193% after CBE in female mice (Figure 3A,B). IL-1β and myeloperoxidase (MPO), an indicator of polymorphonuclear cell infiltration, increased 81% and 355%, respectively, after CBE treatment, indicating inflammation (Figure 3A,C,D). Alda-1 almost completely blocked these inflammatory responses (Figure 3A–D).

In female mice, hepatic smooth muscle α-actin (αSMA) increased 123% after CBE treatment, signifying activation of hepatic stellate cells (HSCs), a critical step that eventually leads to fibrosis (Figure 3A,E). Alda-1 also totally blocked HSC activation after CBE. However, Sirius red-stained fibers did not increase in the liver after CBE treatment, indicating that histologically visible liver fibrosis had not yet occurred at this early stage (Figure 3F).

### 2.3. Chronic Binge Ethanol Causes Mitochondrial Depolarization and Fat Droplet Accumulation and Suppresses Fatty Acid Oxidation in the Liver: Blunting by Alda-1

**Figure 4:**
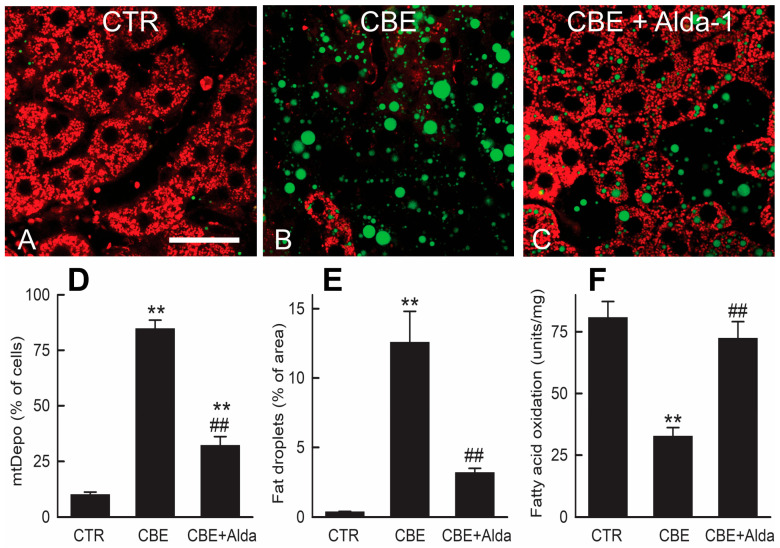
Increased mitochondrial depolarization, steatosis, and inhibited fatty acid oxidation after chronic binge ethanol treatment: suppression by Alda-1. Conditions are as in Figure 1. (**A**–**C**) Mitochondrial polarization status and fat droplets were detected by intravital multiphoton microscopy of TMRM (red) and BODIPY493/503 (green) fluorescence, respectively, at 4 h after control diet with vehicle gavage on day 16 (CTR), EtOH diet with EtOH gavage on day 16 (CBE), or CBE treatment with Alda-1 treatment (CBE + Alda). Representative images are shown. Bar is 20 µm. (**D**) Percentage of hepatocytes with mtDepo; (**E**) BODIPY493/503-positive areas; (**F**) hepatic fatty acid oxidation detected in liver tissue collected at 9 h after CTR diet with vehicle gavage on day 16 (CTR), EtOH diet with EtOH gavage on day 16 (CBE), or CBE treatment with Alda-1 treatment (CBE + Alda) ** *p* < 0.01 vs. CTR. ## *p* < 0.01 vs. CBE. Values are means ± SEM (n = 3–4/per group).

Hepatic mtDepo and fat droplets were revealed with red and green fluorophores tetramethylrhodamine methyl ester (TMRM) and BODIPY493/503, respectively, as visualized by intravital multiphoton microscopy in female mice (Figure 4). Punctate red TMRM fluorescence was observed in nearly all hepatocytes in CTR mice (Figure 4A,D). After CBE treatment, absence of punctate TMRM fluorescence occurred in ~85% of hepatocytes, indicating mtDepo (Figure 4B,D). In Alda-1 treated mice, mtDepo occurred in only 32% hepatocytes after CBE (Figure 4C,D).

In CTR female mice, only a few green-fluorescing microvesicular fat droplets were observed in the liver (Figure 4A). After CBE, fat droplets, both microvesicular and macrovesicular, markedly increased (Figure 4B). BODIPY493/503-positive areas increased from 0.4% in CTR livers to 12.6% after CBE (Figure 4E). Fat droplets were also larger and more abundant in hepatocytes with mtDepo. With Alda-1 treatment, BODIPY493/503-positive areas after CBE treatment decreased to 3.2% (Figure 4C,E).

Mitochondria are the major organelles responsible for fatty acid β-oxidation. We therefore examined hepatic FAO activity. Hepatic FAO was 80.8 units/mg protein in livers from CTR mice, which decreased to 32.7 units/mg after CBE treatment (Figure 4F). With Alda-1 treatment, hepatic FAO activity after CBE was restored to 72.4 units/mg.

### 2.4. Chronic Binge Ethanol Increases Mitophagic Burden: Blunting by Alda-1

**Figure 5:**
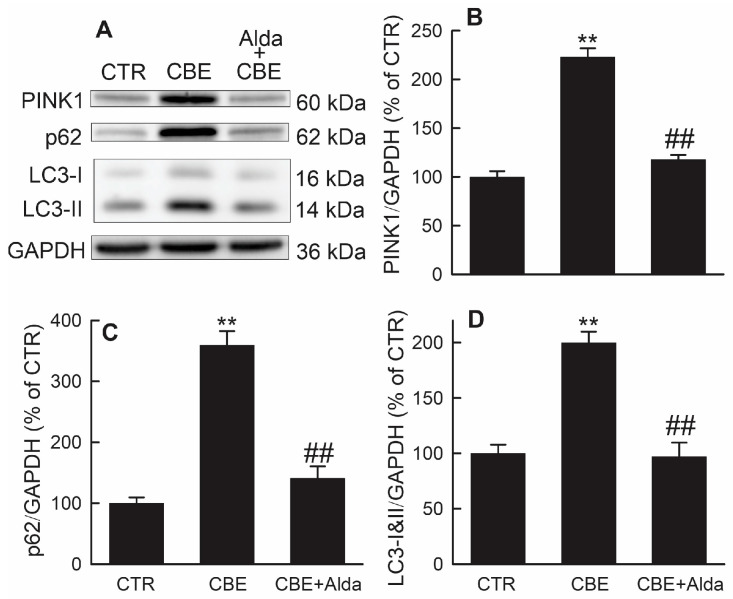
Increased mitophagy after chronic binge ethanol treatment: suppression by Alda-1. Conditions are as in Figure 1. Livers were collected at 9 h after CTR diet with vehicle gavage on day 16 (CTR), EtOH diet with EtOH gavage on day 16 (CBE), or CBE treatment with Alda-1 treatment (CBE + Alda). LC3-I & II, p62, PINK1, and housekeeping protein GAPDH were detected by immunoblotting. (**A**) Representative immunoblot images. (**B**–**D**) Densitometric quantification of immunoblot images. ** *p* < 0.01 vs. CTR. ## *p* < 0.01 vs. CBE. Values are means ± SEM (n = 4/per group).

Our previous study showed that mtDepo after acute EtOH treatment causes Type 2 (damage-associated) mitophagy [24]. In this study we examined whether mitophagic burden also increases after CBE in female mice. mtDepo causes accumulation of PTEN-induced putative kinase 1 (PINK1) on the surface of mitochondria, which triggers mitophagy [34]. After CBE, PINK1 increased by 123% (Figure 5A,B). Other mitophagic markers, ubiquitin-autophagy adaptor 62 (p62) and microtubule-associated protein 1 light chain 3 (LC3)-I & II, increased by 259% and 100%, respectively (Figure 5A,C,D). These alterations signify increased mitophagy. With Alda-1 treatment during CBE, PINK1 and p62 only increased by 17% and 40%, respectively, and LC3-I & II did not increase (Figure 5A,D).

### 2.5. Chronic Binge Ethanol Suppresses Lysosomal Processing: Prevention by Alda-1

**Figure 6:**
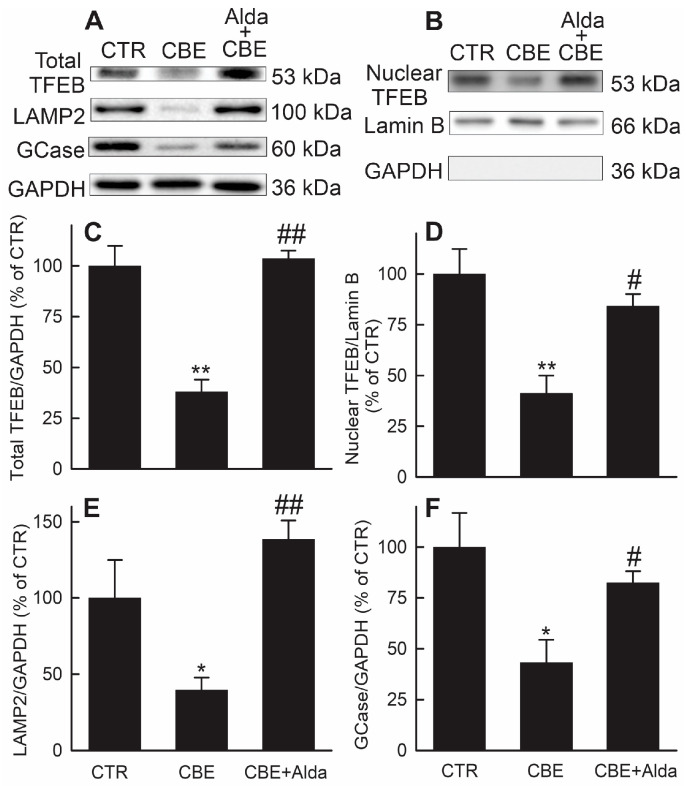
Decrease in hepatic lysosomal biogenesis after chronic binge ethanol treatment: prevention by Alda-1. Conditions are as in Figure 1. Livers were collected at 9 h after CTR diet with vehicle gavage on day 16 (CTR), EtOH diet with EtOH gavage on day 16 (CBE), or CBE treatment with Alda-1 treatment (CBE + Alda). Total and nuclear TFEB, Lamin B, and housekeeping protein GAPDH as well as total LAMP2 and GCase, were detected by immunoblotting. (**A**) Representative immunoblot images of whole extracts. (**B**) Representative immunoblot images of nuclear fractions. (**C**–**F**) Densitometric quantification of immunoblot images. * *p* < 0.05 and ** *p* < 0.01 vs. CTR. # *p* < 0.05 and ## *p* < 0.01 vs. CBE. Values are means ± SEM (n = 4/per group).

Mitophagosomes formed during mitophagy must be processed by lysosomes to remove damaged mitochondria. We therefore examined the lysosomal processing capability after CBE in female mice. Transcription factor-EB (TFEB) is the master regulator of lysosomal gene expression [35,36]. After CBE treatment, total TFEB expression decreased by 62% (Figure 6A,C). With Alda-1 treatment, total TFEB did not decrease. TFEB translocates to nuclei to promote expression of lysosomal proteins [35,36]. Nuclear TFEB decreased 59% after CBE (Figure 6B,D). With Alda-1 treatment, nuclear TFEB only decreased 16%. In these experiments, cytosolic GAPDH was undetectable in nuclear fractions (Figure 6B), demonstrating their high purity. Lysosomal-associated membrane protein 2 (LAMP2), a lysosomal marker [37], decreased 61% after CBE (Figure 6A,E). D-glucosyl-*N*-acylsphingosine glucohydrolase/β-glucocerebrosidase (GCase), another lysosomal protein, also decreased 57% after CBE (Figure 6A,F). Decreases of these lysosomal proteins signify suppressed lysosomal biogenesis. Alda-1 treatment, which recovered TFEB expression, also recovered expression of these lysosomal markers.

### 2.6. Prevention by Alda-1 of Inhibition of Mitochondrial Biogenesis After Chronic Binge Ethanol

**Figure 7:**
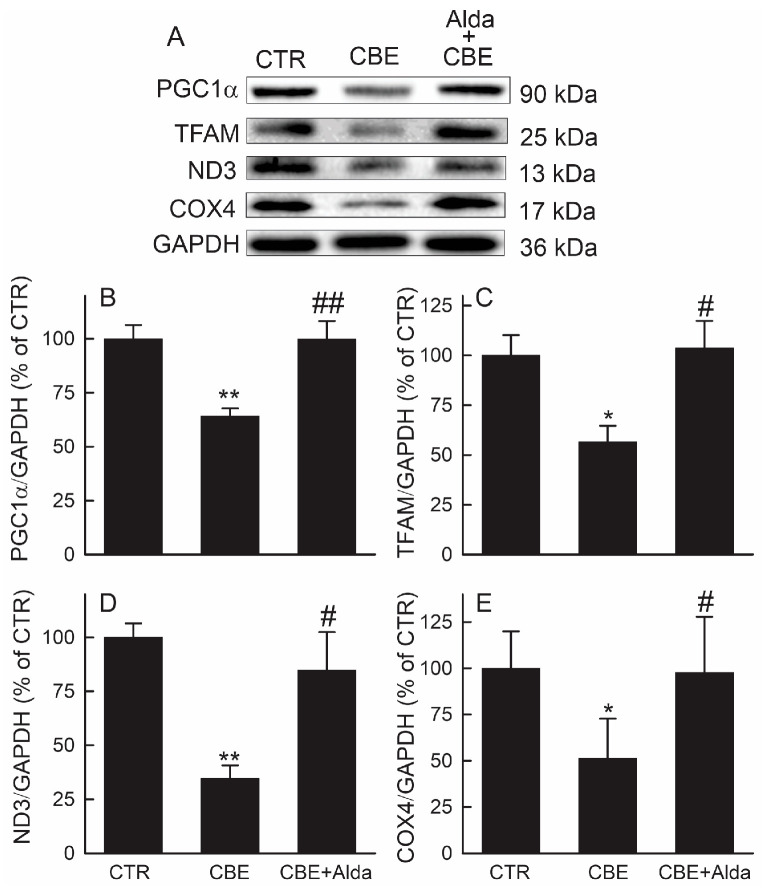
Suppression of hepatic mitochondrial biogenesis after chronic binge ethanol treatment: prevention by Alda-1. Conditions are as in Figure 1. Livers were collected at 9 h after CTR diet with vehicle gavage on day 16 (CTR), EtOH diet with EtOH gavage on day 16 (CBE), or CBE treatment with Alda-1 treatment (CBE + Alda). PGC1α, TFAM, ND3, COX4, and housekeeping protein GAPDH were detected by immunoblotting. (**A**) Representative immunoblot images. (**B**–**E**) Densitometric quantification of immunoblot images. * *p* < 0.05 and ** *p* < 0.01 vs. CTR. # *p* < 0.05 and ## *p* < 0.01 vs. CBE. Values are means ± SEM (n = 4/per group).

Mitochondrial biogenesis (MB) must occur after mitophagy to restore mitochondrial mass and function. Peroxisome proliferator-activated receptor gamma coactivator 1 alpha (PGC1α) is the primary regulator of MB [38,39]. PGC1α expression decreased by 36% after CBE in female mice (Figure 7A,B). Mitochondrial transcription factor-A (TFAM), which controls mtDNA replication and transcription [40], decreased 43% after CBE (Figure 7A,C). Alda-1 blocked alterations of these MB signaling molecules after CBE (Figure 7). MB requires formation of both mtDNA- and nuclear DNA (nDNA)-encoded OXPHOS proteins [38]. NADH dehydrogenase 3 (ND3), a mtDNA-encoded OXPHOS protein, decreased 65% after CBE (Figure 7A,D). Moreover, cytochrome *c* oxidase IV (COX4), a nDNA-encoded OXPHOS protein, decreased by 49% after CBE (Figure 7A,E). After CBE with Alda-1, levels of these OXPHOS proteins were no longer statistically different from CTR.

### 2.7. Release of Mitochondrial Damage-Associated Molecular Patterns After Chronic Binge Ethanol: Inhibition by Alda-1

**Figure 8:**
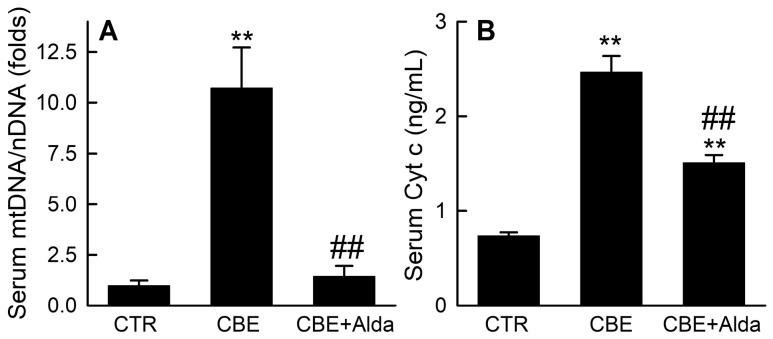
Increases of serum mitochondrial damage-associated molecular patterns after chronic binge ethanol treatment: suppression by Alda-1. Conditions are as in Figure 1, and blood was collected during liver harvesting as in Figure 5, Figure 6 and Figure 7. (**A**) Serum mtDNA/nDNA ratios. (**B**) Serum cytochrome *c*. ** *p* < 0.01 vs. CTR. ## *p* < 0.01 vs. CBE. Values are means ± SEM (n = 4/per group).

Impaired lysosomal processing of mitophagosomes may lead to release of mtDAMPs. We examined mtDAMPs in sera with and without CBE treatment in female mice (Figure 8). After CBE, the serum mtDNA/nDNA ratio increased 970% compared to that in CTR mice (Figure 8A). In Alda-1-treated mice, the serum mtDNA/nDNA ratio did not increase significantly after CBE. Cytochrome *c*, another mtDAMP, in the serum increased from a basal value of 0.74 ng/mL to 2.47 ng/mL after CBE (Figure 8B). With Alda-1 treatment, cytochrome *c* only increased to 1.51 ng/mL.

### 2.8. mtDNA Activates Macrophages In Vitro

**Figure 9:**
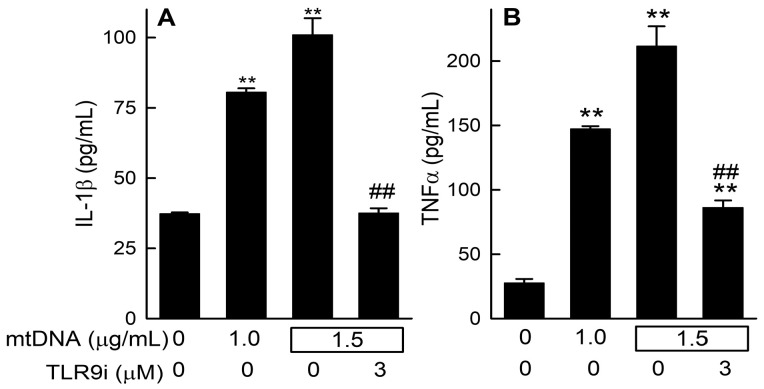
Mitochondrial DNA activates macrophages in vitro. RAW264.7 macrophages were cultured with mouse liver mtDNA (1–1.5 µg/mL), and AT791 (3µM), a TLR9 inhibitor (TLR9i), was added to some dishes. Interleukin-1β (IL-1β, (**A**)) and tumor necrosis factor-α (TNFα, (**B**)) in culture medium were detected using ELISA kits. ** *p* < 0.01 vs. CTR. ## *p* < 0.01 vs. mtDNA 1.5 µg/mL. Values are means ± SEM (n = 3/per group).

To explore whether mtDAMPs have proinflammatory effects, we examined whether the mtDAMP, mtDNA, activates RAW264.7 macrophages in vitro. After incubation with 1.0 and 1.5 µg/mL of mtDNA, expression of the proinflammatory cytokine interleukin-1β (IL-1β) increased from a basal value of 37 pg/mL to 80 and 101 pg/mL, respectively (Figure 9A). Toll-like receptor-9 (TLR9) is a pattern recognition receptor that recognizes mtDNA, as well as foreign DNA from bacterial and viruses [41]. In the presence of the TLR9 inhibitor AT791, 1.5 µg/mL of mtDNA did not increase IL-1β.

After incubation with 1.0 and 1.5 µg/mL of mtDNA, tumor necrosis factor-alpha (TNFα), another proinflammatory cytokine, increased from a basal value of 27 pg/mL to 147 and 212 pg/mL, respectively (Figure 9B). In the presence of AT791, 1.5 µg/mL of mtDNA only increased TNFα to 86 pg/mL, indicating that mtDNA activates macrophages through binding to TLR9.

### 2.9. mtDNA Activates Hepatic Stellate Cells In Vitro

**Figure 10:**
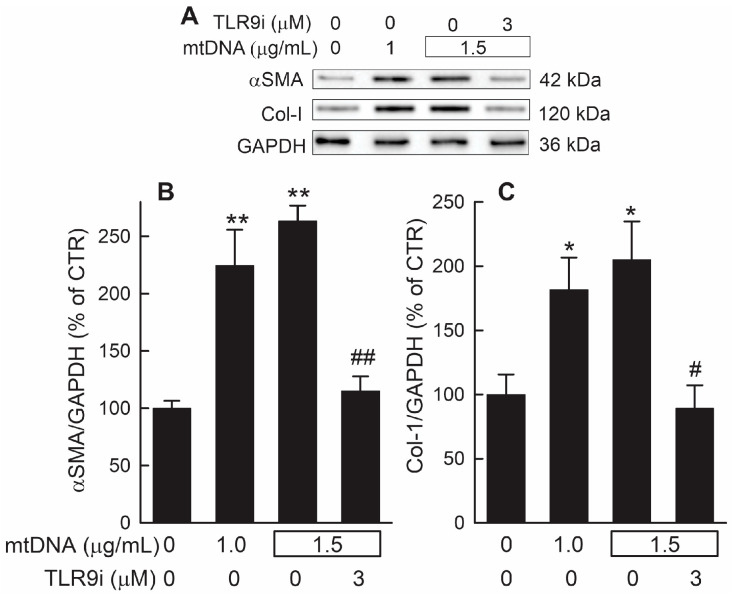
mtDNA activates hepatic stellate cells in vitro. hTERT-HSC cells were cultured with mouse liver mtDNA (1–1.5 µg/mL, (**A**–**C**)). AT791 (3 uM), a TLR9 inhibitor (TLR9i), was added to some dishes. αSMA, Col-1, and housekeeping protein GAPDH in cell lysates were detected by immunoblotting. (**A**) Representative immunoblot images. (**B**,**C**) Densitometric quantification of immunoblot images. * *p* < 0.05 and ** *p* < 0.01 vs. CTR. # *p* > 0.05 and ## *p* < 0.01 vs. mtDNA 1.5 µg/mL. Values are means ± SEM (n = 3/per group).

To explore if mtDAMPs have profibrotic effects, we examined whether mtDNA activates immortalized human HSC (hTERT-HSC) in vitro. After incubation with 1.0 and 1.5 µg/mL of mtDNA, expression of aSMA increased 124% and 163%, respectively (Figure 10A,B), and expression of collagen-1 (Col-1) increased 82% and 105%, respectively (Figure 10A,C). In the presence of the TLR9 inhibitor AT791 and 1.5 µg/mL of mtDNA, αSMA and Col-1 did not increase.

## 3. Discussion

### 3.1. Dysregulated Mitophagic Processing Occurs After Chronic Binge Ethanol Treatment

Widespread mtDepo occurs after an acute, single dose of EtOH [20]. After EtOH exposure, damaged/depolarized mitochondria need to be removed/repaired to restore mitochondrial homeostasis and cell health. One of these recovery processes is mitophagy. Mitophagy removes damaged, effete, and superfluous mitochondria. Pertinent to this study, mitochondrial damage and depolarization initiate Type 2 mitophagy with mtDepo causing PINK1 accumulation on mitochondria that promotes Parkin binding, recruitment of autophagy receptor proteins like p62, and association with LC3-containing membranes. The result is formation of a mitophagosome enveloping the target mitochondrion [42,43].

Our recent study showed that mtDepo after acute EtOH activates Type 2 mitophagy in GFP-LC3 transgenic mice, as evidenced by markedly increased GFP-LC3 puncta predominantly in hepatocytes with mtDepo [24]. Blockade of mtDepo by either Alda-1 (which decreases AcAld) or tacrolimus (which does not decrease AcAld) also decreased mitophagy [24]. Lysosomal processing of mitophagosomes also increased when GFP-LC3 transgenic mice were exposed to acute EtOH. Although expression of TFEB, the master regulator of lysosomal biogenesis [35,36], did not increase after acute EtOH, TFEB nonetheless translocated from cytosol to nuclei [24]. TFEB nuclear translocation was accompanied by increased expression of lysosomal markers and co-localization of GFP-LC3 puncta with rhodamine dextran-labeled lysosomes [44], documenting lysosomal processing of mitophagosomes [24]. Increased lysosomal processing removes mitochondria damaged by acute EtOH, an apparent protective mechanism. Mitochondrial remodeling and biogenesis possibly also occur subsequently to restore mitochondrial mass and function. Mitochondrial polarization status recovers in 85% of hepatocytes in 24 h after acute EtOH treatment [20]. In association with these adaptive responses to acute EtOH, microvesicular steatosis occurs, but liver injury was very mild (ALT release ~100 U/L, ~2% cell death), and inflammation was not observed. These changes represent simple steatosis [20]. mtDepo and consequent ATP depletion can decrease fatty acid β-oxidation, thus leading to steatosis. mtDepo may also cause necrosis as well as apoptosis through the intrinsic (mitochondrial) pathways [45]. However, liver injury is very mild after acute EtOH, possibly because mtDepo is transient under such circumstance [20].

The transition from simple steatosis to steatohepatitis and fibrosis is a critical step in the progressioin of ALD, which eventually leads to detrimental outcomes in advanced stages [1,2]. However, the mechanism(s) of this transition remain unclear. Abnormalities of mitochondria are one of the earliest manifestations of hepatocyte injury by alcohol. In this study, we aimed to determine how adaptive mitochondrial alterations that enhance detoxifying ethanol (EtOH) metabolism after acute treatment transition into maladaptive responses that contribute to ALD progression in a CBE model, which is widely recognized for inducing steatosis, liver injury, and inflammation in mice [27,46,47]. We showed that, unlike the minimal liver injury and absence of inflammation observed after acute EtOH exposure, CBE treatment produced a marked increase in serum ALT (~350 U/L) that was accompanied by micro- and macrovesicular steatosis, necrosis, apoptosis, inflammasome activation, proinflammatory cytokine production, and leukocyte infiltration (Figure 2 and Figure 3). Although histologically visible fibrosis was not evident after CBE, αSMA, a marker of HSC activation, increased (Figure 3). Together, these results confirm that CBE causes active steatohepatitis with profibrotic activation of HSCs, a critical step leading to fibrosis. Thus, this CBE model differs in important ways from the acute EtOH model.

What is the mechanism underlying the transition from simple steatosis after acute ethanol to active steatohepatitis after CBE? Similar to acute EtOH, mtDepo occurred after CBE, and this effect was blunted by the ALDH2 activator, Alda-1, demonstrating that AcAld-driven mtDepo also occurs after CBE (Figure 4). After CBE, PINK1, p62 and LC3-I/II increased, signifying increased mitophagy (Figure 5). Although both acute EtOH and CBE caused mtDepo and increased mitophagic burden, lysosomal biogenesis and the processing of mitophagosomes differed markedly between the two treatments. Unlike after acute EtOH, total TFEB expression and nuclear TFEB markedly decreased after CBE, which was accompanied by substantially lower lysosomal protein markers (LAMP2 and GCase) (Figure 6) [37]. Alda-1 treatment restored expression of TFEB and its nuclear localization, as well as the expression of lysosomal protein markers, indicating that prolonged exposure to AcAld in CBE leads to suppression of lysosomal biogenesis and function (Scheme 1B). Consistently, others also show that TFEB decreases after chronic EtOH treatment in mice and in patients with alcohol-associated hepatitis, indicating suppressed lysosomal biogenesis after chronic EtOH [48,49]. In cultured Huh 7.5 liver cells, AcAld directly impairs TFEB nuclear translocation by increasing acetylation of α-tubulin, which likely contributes to EtOH-induced suppression of lysosomal degradation of HIV proteins [50]. Moreover, increased TFEB acetylation markedly suppresses its transcriptional function and decreases lysosomal biogenesis [51]. Therefore, AcAld can also directly impair the function of TFEB.

### 3.2. Proinflammatory/Profibrotic Mitochondrial Damage-Associated Molecular Patterns Are Released After Chronic Binge Ethanol Treatment

Why does suppressed lysosomal biogenesis increase liver injury, inflammation, and HSC activation? A possible explanation is that increased mtDepo-driven mitophagic burden with decreased lysosomal biogenesis limits lysosomal processing of mitophagosomes after CBE. Consequently, mtDAMPs of depolarized/damaged mitochondria are not degraded completely and are instead released into the cytosol and extracellular space [13,52,53]. mtDAMPs include many molecules, such as mtDNA, ATP, cytochrome c, *N*-formyl peptides, cardiolipin, and TFAM. The release of mtDAMPs has been widely reported in both human and animals [54,55], but pathways for release are incompletely characterized. Previous studies demonstrated that mitochondrial DNA (mtDNA) can be released in microvesicles, likely as a mechanism during CBE [56]. Future studies will be needed to determine whether exocytosis of unprocessed autophagosomes (fusion of autophagosomes with the plasma membrane) can account for microvesicular release of mtDNA and other mtDAMPs after chronic EtOH treatment

mtDAMPs initiate innate and adaptive immune responses by binding to cellular receptors, such as TLR9 for mtDNA, to cause NLRP3 inflammasome activation in immune cells [52]. Many mtDAMPs are cytotoxic, proinflammatory, and profibrotic. Therefore, they may contribute to ALD pathogenesis, as we proposed previously [13,53]. In support of this hypothesis, serum mtDNA and cytochrome c markedly increased after CBE, which Alda-1 partially or completely prevented (Figure 8). In vitro, mtDNA activated macrophages to release proinflammatory cytokines and HSC to express fibrogenic markers (αSMA and collagen-1) through TLR9 (Figure 9 and Figure 10). Together, these findings support the conclusion that mtDAMPs like mtDNA are proinflammatory and profibrotic and thus capable of promoting the progression of ALD (Scheme 1B).

Previous studies show that treatment with quercetin to activate mitophagy and overexpression of TFEB to increase lysosomal processing are protective in animal models of ALD and metabolic dysfunction-associated steatotic liver disease [49,57,58]. Our data also indicate that impaired lysosomal processing of autophagosomes and inadequate clearance of damaged mitochondria, not mitophagic sequestration per se, promote mtDAMP release and tissue injury after CBE treatment. Thus, this dysregulated mitophagy and lysosomal processing likely act as a tipping point that changes adaptive mitochondrial alterations after EtOH to maladaptive responses and progression of ALD from simple steatosis to steatohepatitis and fibrosis (Scheme 1B). Additionally, chronic exposure to AcAld may also directly cause cell damage and contribute to inflammation and impairment of lysosomal processing.

### 3.3. Suppressed Mitochondrial Biogenesis After Chronic Binge Ethanol Inhibits Recovery of Mitochondrial Function

Although mitophagy is considered as a cell-protective response by removing damaged, effete, and superfluous mitochondria [42], mitochondrial biogenesis (MB) must also occur to restore mitochondria mass and maintain mitochondrial homeostasis after mitophagy and lysosomal processing. Impairment of mitochondrial homeostasis contributes to various hepatic pathologies, including steatosis, cell death, inflammation, and fibrosis [59,60]. After acute EtOH treatment, mitochondrial polarization recovers in ~85% of hepatocytes in 24 h [20]. A possible mechanism for recovery of mitochondrial polarization is MB. Interestingly, previous studies showed that after acute EtOH treatment, mtDNA in multiple organs including the liver markedly decreases and then recovers to or above the basal values in 24 h, consistent with loss of mitochondrial mass by mitophagy and recovery by MB [61,62]. However after CBE, PGC1α (master regulator of MB), TFAM (transcription factor controlling mtDNA replication and transcription), and nDNA- and mtDNA-encoded OXPHOS proteins (COX4 and ND3) decreased, documenting suppressed MB after CBE (Figure 7) [38,39,40]. The mechanism of MB inhibition after CBE remains unclear. Interestingly, a transcriptional characterization study revealed that PGC1α is a TFEB targeted gene and that TFEB drives *Pgc1α* gene expression in adipocytes to protect against diet-induced metabolic dysfunction [63]. Additionally, TFEB deficiency completely blocks *Pgc1α* gene expression in muscle cells [64]. TFEB-driven PGC1α expression possibly represents an important mechanism coordinating MB to recover the mitochondrial mass and function lost by mitophagy and lysosomal processing. However, after CBE, decreased TFEB due to prolonged exposure to EtOH/AcAld possibly decrease PGC1α expression, thus suppressing MB and the recovery of mitochondrial mass and function after CBE, an additional mechanism promoting progression of ALD (Scheme 1B).

In conclusion, our study showed that after CBE, AcAld-driven mtDepo still occurs (Figure 4), but co-ordination of mitophagy and lysosomal processing becomes dysregulated (Figure 5 and Figure 6), leading to release of mtDAMPs that promote inflammatory and fibrotic processes (Figure 8, Figure 9 and Figure 10) (Scheme 1B). In addition, MB becomes suppressed, thus inhibiting recovery of mitochondrial functions like OXPHOS and FAO (Figure 7) (Scheme 1B). Together, dysregulation of mitophagy-lysosomal processing and mitochondrial homeostasis likely promotes the transition from adaptive mitochondrial responses for more rapid detoxifying metabolism of EtOH to a maladaptation leading to mtDAMP release as a critical contributor to the multi-hit pathogenesis of ALD (Scheme 1A,B). Understanding of this mitochondria-related pathogenesis may lead to identification of new therapeutic strategies. Overall, our data demonstrate that AcAld plays a crucial role driving development of maladaptation. AcAld levels markedly increased after CBE treatment, but this effect was blunted by the ALDH2 activator Alda-1, which reduced AcAld accumulation (Figure 1). Importantly, Alda-1 decreased or reversed CBE-induced mtDepo, dysregulation of mitophagic lysosomal processing, mtDAMP release, and MB suppression (Figure 4, Figure 5, Figure 6, Figure 7 and Figure 8), thus attenuating steatohepatitis and HSC activation (Figure 2 and Figure 3). Thus, drugs that enhance detoxifying aldehyde metabolism, lysosomal processing, and MB, as well as drugs that neutralize mtDAMPs in the blood or blockade of their interactions with inflammatory and fibrotic effector cells, are promising to prevent progression of ALD, onset of end-stage liver disease, and development of liver cancer.

## 4. Materials and Methods

### 4.1. Materials

The sources of all chemicals, antibodies, and other reagents are listed in Table S1 in the Supplementary Materials.

### 4.2. Synthesis of Alda-1

Alda-1, an ALDH2 activator [65], was synthesized as described previously [66]. The structure of the product was verified by NMR spectroscopy, and the purity of the product was >99% as assessed by HPLC chromatography [66].

### 4.3. Animals and Chronic Binge Ethanol Treatment

**Scheme 2:**
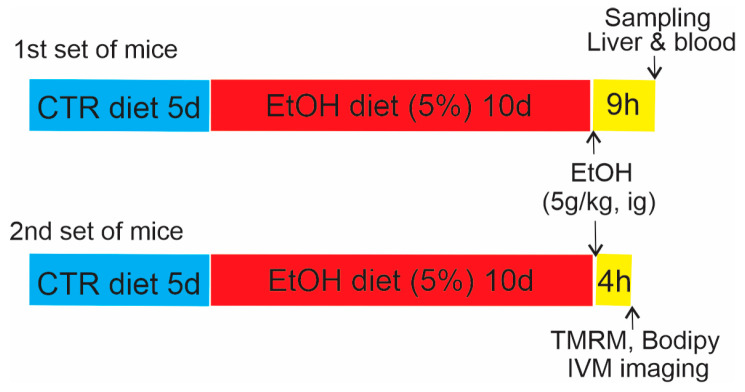
Diagram of the experimental procedures. IVM, intravital multiphoton microscopy.

*C57BL/6* mice (both male and female, 10–11 wks old, Charles River, Charleston, SC, USA) were subjected to CBE treatment according to the protocol of the National Institute on Alcohol Abuse and Alcoholism CBE mouse model described previously [46]. Numbers of mice used are indicated in the figure legends. In brief, mice were fed a CTR liquid diet (Table S1) for 5 d to allow mice to adapt to liquid diet feeding, followed by a liquid diet containing 5% (*v*/*v*) EtOH (Table S1) for 10 d. On Day 16, mice were gavaged with EtOH (5 g/kg body weight as 31.8% EtOH in saline) once in the morning [46]. CTR mice were fed CTR liquid diet throughout the whole feeding period and then gavaged with one dose of sucrose-dextrin (Table S1) at equal calories and equal volume as EtOH on the last day [46]. Alda-1 was dissolved at 20 mg/mL in a mixture of Neobee and DMSO (7:3) and injected subcutaneously daily at 20 mg/kg body weight during EtOH diet feeding and at 50 mg/kg 30 min before gavage of EtOH on Day 16. The CTR group received equal volumes of the vehicle. Two sets of mice were used. The first set was used to collect blood and liver tissue under ketamine/xylazine anesthesia (90 mg/kg and 10 mg/kg, i.p., respectively) at 9 h after gavage on Day 16 (Scheme 2). Serum and liver tissue were stored at −80 °C until use. The second set was used to detect mitochondrial polarization status and fat accumulation by intravital multiphoton microscopy, as described in Section 4.9 (Scheme 2). Major findings were confirmed in both males and females, but females were used for most mechanistic studies, since both rodent and human females are reported to be more susceptible to ALD [67,68]. All animals were given humane care in compliance with institutional guidelines using protocols pre-approved by the Institutional Animal Care and Use Committee (protocol AR# 2019-00861, date of approval: 30 January 2020).

### 4.4. Measurement of Alanine Aminotransferase in Sera

Serum ALT activity was determined using a commercial kit (Table S1) according to the manufacturer’s instructions.

### 4.5. Histology and Detection of Fibrosis on Liver Sections

After tissue harvest, parts of livers were fixed in 10% neutralized formaldehyde for 24–48 h. Deparaffinized liver sections (5 µm) were stained with hematoxylin/eosin (H&E) for histological examination. Deparaffinized liver sections were also stained with Sirius red/Fast green to reveal liver fibrosis [69,70]. Liver images were acquired using a Zeiss AX10 microscope (White Plains, NY, USA) with 10× and 20× objective lenses.

### 4.6. Isolation of Hepatic Nuclear Fractions

TFEB, the master regulator of lysosomal biogenesis, translocates to nuclei to promote expression of lysosomal proteins [35,36]. To assess TFEB in nuclear fractions, liver tissue was homogenized, and nuclear fractions were isolated using a Cytosol and Nuclear Isolation Kit (Table S1) according to the manufacturer’s instructions. TFEB and lamin B, a housekeeping protein for the nuclear fraction, were detected by immunoblotting. GAPDH, a protein that mainly exists in cytosol, was also detected to verify the purity of nuclear fractions.

### 4.7. Measurement of Mitochondrial Damage-Associated Molecular Patterns in Sera

To examine whether CBE treatment increases the release of mtDAMPs, we measured mtDNA and cytochrome *c* in sera. Total DNA was isolated from sera using the DNeasy Blood and Tissue Kit (Table S1). mtDNA and nuclear DNA (nDNA) were then measured by quantitative polymerase chain reaction (qPCR) [71,72,73]. mtDNA content was determined as mtDNA-encoded NADH dehydrogenase-1 and normalized against the nuclear-encoded 18S RNA gene, as described previously [71,72,73]. Serum cytochrome *c* was detected using an ELISA kit (Table S1) according to the manufacturer’s instructions.

### 4.8. Hepatic Fatty Acid Oxidation

FAO capacity in liver tissue was detected using a Fatty Acid Oxidation Assay kit (Table S1) according to the manufacturer’s instruction. Briefly, liver tissue (~5 mg) was homogenized in 500 µL ice-cold lysing solution from the kit. After centrifugation at 14,000 rpm and 4 °C for 5 min, the supernatants were collected, and protein was determined using a Pierce BCA protein assay kit (Table S1). Supernatants with 20 µg protein were mixed with 50 µL FAO assay solution containing 2.5 µL octanoyl-CoA and incubated at 37 °C for 30 min. The optical density of formazan at 492 nm was measured using a Spectramax m2 micro plate reader (Molecular Devices, San Jose, CA, USA). The FAO activity was normalized by protein concentration in each sample.

### 4.9. Detection of Mitochondrial Depolarization and Fat Droplets in Mouse Livers by Intravital Multiphoton Microscopy

At 4 h after gavage of EtOH on day 16, BODIPY493/503 (Table S1) was used to label fat droplets [20], and TMRM, a membrane-permeant cationic fluorophore that accumulates in mitochondria driven by mitochondrial ΔΨ, was used to reveal mitochondrial polarization status (Scheme 2), since the intensity of TMRM fluorescence is proportional to negative inside ΔΨ [74]. Although TMRM uptake into mitochondria is driven by both plasmalemmal and mitochondrial ΔΨ, a variety of experimental data indicate that loss of TMRM uptake after EtOH is predominantly due to collapse of the mitochondrial ΔΨ (mtDepo). The plasmalemmal ΔΨ is −30–35 mV, whereas the inner mitochondrial ΔΨ is −150–180 mV [75,76,77]. Compared to the surface to volume ratio of hepatocytes, mitochondria have a much larger inner membrane surface to volume ratio due to invaginating cristae membranes. Consequently, cationic fluorophores like rhodamine 123 and TMRM equilibrate across the mitochondrial inner membrane much more rapidly than across the plasma membrane. Previous studies showed that after mtDepo, rapid release of these dyes from mitochondria leads to a flush of increased fluorescence in the cytosol as observed in vitro in isolated hepatocytes as well as after in vivo EtOH treatment (see Figure 2 of [78] and Figure 2B of [20]). Over time, this flush dissipates as the fluorophores permeate across the plasma membrane. If the plasma membrane was depolarizing instead of the mitochondrial membrane, then a flush of cytosolic fluorescence would not occur. Additionally, mitochondrial NAD(P)^+^ increases in vivo after EtOH only in mitochondria that have released TMRM [20]. This is due to the NADH-oxidizing respiratory burst after mtDepo. Therefore, for this and other reasons, TMRM release after EtOH reliably reflects mtDepo rather than collapse of plasmalemmal ΔΨ.

Briefly, under ketamine and xylazine (90 mg/kg and 10 mg/kg, i.p.) anesthesia, a 20 G catheter was inserted into the trachea and connected to a small animal respirator. TMRM (1.1 µmol/mouse; Table S1) and BODIPY493/503 (75 µg/mouse) were infused slowly into the carotid artery over 10 min [79]. Laparotomized mice were placed on the stage of microscope in a prone position, and intravital imaging was performed with an Olympus FluoView 1200 MPE multiphoton microscope (Evident, Center Valley, PA, USA) equipped with a Spectra Physics Mai Tai Deep Sea tunable multiphoton laser (Newport, Irvine, CA, USA), as described previously [79]. Using 920 nm multiphoton excitation, fluorescence was imaged simultaneously 10 to 50 µm deep from the liver surface through 575–630 nm and 495–540 nm band pass emission filters for TMRM and BODIPY493/503, respectively. The respirator was turned off for 5–10 s during imaging to eliminate breathing artifacts. Ten or more random images were collected for each mouse.

Punctate TMRM red fluorescence represents cells with polarized mitochondria whereas a dimmer diffuse cytosolic fluorescence signifies mtDepo. Hepatocytes with mtDepo were counted in ~10 images per mouse, and the percentage of cells with mtDepo was calculated. The areas of green BODIPY493/503-positive lipid droplets were quantified from 10 images per mouse using ImageJ software version Fiji-2.14.0.

### 4.10. Effects of Mitochondrial DNA on Immortalized Human Hepatic Stellate Cells and Macrophages In Vitro

Activation of HSC is a critical step in liver fibrosis. To examine whether mtDNA causes HSC activation in vitro, immortalized human HSC (hTERT-HSC), a cell line often used to study the cell biology of human HSC in vitro [80], were cultured in DMEM high glucose medium (Table S1) with 10% fetal bovine serum at 5% carbon dioxide and 37 °C for 24 h until they reached ~70% confluence. The medium was then changed to DMEM with 0.5% FBS with or without TLR9 inhibitor AT791 (3µM) (Table S1). mtDNA was isolated and purified from the liver of naive *C57BL/6* mice using a mitochondrial DNA isolation kit (Table S1) according to the manufacturer’s instructions and then stored at −80 °C until use. Mouse hepatic mtDNA was added to the culture medium at final concentrations of 1 and 1.5 µg/mL 1 h after addition of AT791. hTERT-HSC were collected 48 h after addition of mtDNA and lysed with ice-cold lysis buffer (Table S1) [81]. Cell lysates were used for immunoblotting of αSMA and Col-1. To examine if mtDNA has proinflammatory effects, RAW264.7 cells, a widely used macrophage cell line (Table S1), were cultured and treated as described above for hTERT-HSC. TNFα and IL-1β in culture medium were detected using ELISA kits (Table S1) according to the manufacturer’s instructions.

### 4.11. Immunoblotting

Proteins of interest in liver tissue extracts, nuclear fractions, and cell lysates were detected by immunoblotting, as described previously [82]. Primary and secondary antibodies are listed in Table S1. Immunoblots were incubated with primary antibodies at a dilution of 1:1000 to 1:3000 overnight at 4 °C, then with the secondary antibodies at a dilution of 1:10,000 at RT for 1 h, and finally with the SuperSignal Reagent (Table S1) for about 1 min. Images of blots were captured using the Chemidoc Touch Imaging System (Bio-Rad, Hercules, CA, USA). Intensities of bands were quantified using NIH ImageJ software.

### 4.12. Statistical Analysis

All groups were compared using ANOVA followed by Student/Newman/Keuls post hoc test, a stepwise multiple comparison procedure used to determine whether sample means are significantly different from each other when there are three or more groups. Statistical analysis was performed using the Sigmaplot software version 12.5. Values are means ± SEM. Differences were considered significant at *p* < 0.05. Group sizes are shown in the figure legends.

## Data Availability

The raw data supporting the conclusions of this article will be made available by the authors on request.

## Supplementary Materials

The following supporting information can be downloaded at: https://www.mdpi.com/article/10.3390/ijms262311608/s1.

## Abbreviations

The following abbreviations are used in this manuscript:

AcAld	acetaldehyde
ADH	alcohol dehydrogenase
ALDH2	aldehyde dehydrogenase-2
ALD	alcohol-associated liver disease
Alda-1	N-(1,3-benzodioxol-5-ylmethyl)-2,6-dichlorobenzamide
ALT	alanine aminotransferase
CBE	chronic binge ethanol treatment
CC3	cleaved caspase-3
Col-1	collagen-1
COX4	cytochrome c oxidase IV
CTR	control
CYP2E1	cytochrome-P450 2E1
DMSO	dimethyl sulfoxide
EtOH	ethanol
FAO	fatty acid oxidation
GCase	β-glucocerebrosidase/D-glucosyl-*N*-acylsphingosine glucohydrolase
GAPDH	glyceraldehyde-3-phosphate dehydrogenase
GPR91	G-protein coupled receptor-91
HSC	hepatic stellate cell
hTERT-HSC	immortal human HSC
IL-1β	interleukin-1β
JO2	oxygen consumption
LAMP2	lysosomal-associated membrane protein-2
MAA	malondialdehyde-acetaldehyde adducts
MAP1LC3/LC3	microtubule-associated protein 1 light chain 3
MB	mitochondrial biogenesis
mtDAMPs	mitochondrial damage-associated molecular patterns
mtDepo	mitochondrial depolarization
mtDNA	mitochondrial DNA
MDVs	mitochondria-derived vesicles
NAD^+^	nicotinamide adenine dinucleotide
ND3	NADH dehydrogenase-3
nDNA	nuclear DNA
NLRP3	NOD-like receptor protein 3
OXPHOS	oxidative phosphorylation
p62	ubiquitin-autophagy adaptor 62
PI3K	Class III phosphoinositide 3-kinase
PGC1α	peroxisome proliferator-activated receptor gamma coactivator-1 alpha
PINK1	PTEN-induced putative kinase 1
qPCR	quantitative polymerase chain reaction
SIAM	swift increase in alcohol metabolism
αSMA	smooth muscle α-actin
TFAM	mitochondrial transcription factor-A
TFEB	transcription factor-EB
TLR9	toll-like receptor 9
TMRM	tetramethylrhodamine methylester
TNFα	tumor necrosis factor-α
ΔΨ	membrane potential
