# Supplementary material for: Role of Acetaldehyde and Dysregulated Mitophagic Lysosomal Processing in Chronic-Binge Ethanol-Induced Liver Injury
Source: Int J Mol Sci. 2025 Nov 29;26(23):11608. doi: 10.3390/ijms262311608 (PMC12692339; doi:10.3390/ijms262311608)
Supplement: Supplementary file 1 [file ijms-26-11608-s001.zip › ijms-3887641-supplementary.pdf]

Supplemental Information

Role of Acetaldehyde and Dysregulated Mitophagic Lysosomal Processing in Chronic-Binge Ethanol-Induced Liver Injury

Devadoss J. Samuvel [1], Emory Foerster [1], Li Li [1], Amir K. Richardson [1], Patrick M. Wooster [1,†], John J. Lemasters [1,2] and Zhi Zhong [1,*]

[1] Department of Drug Discovery & Biomedical Sciences, Medical University of South Carolina,
Charleston, SC 29425, USA; samuvel@musc.edu (D.J.S.); emoryafoerster@gmail.com (E.F.); lilili@musc.edu (L.L.); richaami@musc.edu (A.K.R.); woster@musc.edu (P.M.W.); lemaste@musc.edu (J.J.L.)
[2] Department of Biochemistry & Molecular Biology, Medical University of South Carolina, Charleston, SC 29425, USA
* Correspondence: zhong@musc.edu; Tel.: +1-(843)-792-2163
† Deceased. .

Table of Contents

Pages	Contents
2 - 4	Table S1
5	Figure S1
6	Figure S2

Table S1. Sources of Reagents and Cell Lines		
Items	***Sources***	***Catalog #***
**Alanine transaminase kit**	Pointe Scientific, Uncoln Park, MI	A7526-150
**AT791**	MedChem Express, Monmouth Junction, NJ	HY-124603
**ATP synthase-β antibody**	Abcam, Waltham, MA	ab14730
**BODIPY493/503**	Fisher Scientific, *Waltham, MA*	D3922
**Cleaved caspase-3 antibody**	Cell Signaling Technology, Danvers, MA	9661
**Control liquid diet**	Bio-serv, Flemington, NJ	F1259SP
**Cytochrome c oxidase subunit-4 antibody**	Santa Cruz Biotech., Santa Cruz, CA	Sc-69360
**Cytochrome c Quantikine ELISA Kit**	R&D systems, Minneapolis, MN	MCTC0
**Cytosol and nucleus isolation kit**	Invent Biotech, Plymouth, MN	NT-032
**Dimethyl sulfoxide**	Sigma-Aldrich, St. Louis, MO	D2650
**DMEM medium**	Fisher Scientific, *Waltham, MA*	11-995-065
**ELISA MAX™ standard set for mouse IL-1β**	BioLegend, San Diego, CA	432601
**ELISA MAX™ standard set for mouse TNFα**	BioLegend, San Diego, CA	430901
**ECL chemiluminescence kit**	Pierce Biotec., Rockford, IL	34075
**Ethanol liquid diet**	Bio-serv, Flemington, NJ	F1258SP
**Fatty acid oxidation assay kit**	Assay Genie, Dublin, Irland	BR00001
**β-glucocerebrosidase antibody**	Proteintech, Rosemont, IL	27972-1-AP
**Glyceraldehyde 3-phosphate dehydrogenase antibody**	Cell Signaling Technology, Danvers, MA	2118
**Harris hematoxylin**	Sigma-Aldrich, St. Louis, MO	HHS32
**Immortalized human hepatic stellate cells-SV40T**	Creative Bioarray, Shirley, NY	CSC-12096Z
**Interleukin-1β antibody**	Santa Cruz Biotech., Santa Cruz, CA	sc-7884
**IQ-Sybr green mix**	Bio-Rad Laboratories, Hercules, CA	1708882
**Lamin B1 antibody**	Santa Cruz Biotech., Santa Cruz, CA	sc-6216
**LC3A/B antibody**	Cell Signaling Technology, Danvers, MA	4108

**Lysosome-associated membrane protein 2 antibody**	Proteintech, Rosemont, IL	66301-1-Ig
**Maltose dextrin**	Dyets, Bethlehem, PA	402850
**Myeloperoxidase antibody**	DAKO Corp., Carpinteria, CA	A0398
**Mitochondrial DNA isolation kit**	Abcam, Waltham, MA	ab65321
**Mitochondrial transcription factor-A antibody**	GenWay Biotech, San Diego, CA	GWB-22C6C2
**NADH dehydrogenase subunit 3 antibody**	Santa Cruz Biotech., Santa Cruz, CA	Sc-26760
**10% Neutral buffered formalin**	Leica Biosystems, Buffalo grove, IL	3800598
**NOD-like receptor protein 3 antibody**	Abcam, Waltham, MA	ab263899
**Peroxisome proliferator-activated receptor-γ coactivator-1α antibody**	Santa Cruz Biotech., Santa Cruz, CA	Sc-13067
**Peroxidase AffiniPure goat anti-rabbit IgG**	Fisher Scientific, *Waltham, MA*	NC9611376
**Peroxidase AffiniPure goat anti-mouse IgG**	Fisher Scientific, *Waltham, MA*	NC9491974
**Pierce BCA protein assay kit**	Pierce Biotec., Rockford, IL	PI23227
**PTEN induced putative kinase 1 antibody**	Santa Cruz Biotech., Santa Cruz, CA	sc-517353
**Raw 264.7 cells**	ATCC, Manassas, VA	TIB-71
**RIPA lysis buffer**	Fisher Scientific, *Waltham, MA*	AAJ63324EQE
**Serum DNA isolation kit**	BioChain, Newark, CA	K5018100
**α-Smooth muscle actin antibody**	Cell Signaling Technology, Danvers, MA	14968
**SQSTM1/p62 Antibody**	Cell Signaling Technology, Danvers, MA	5114
**Transcription factor-EB antibody**	Bethyl laboratories, Montgomery, TX	A303-673A

**Tetramethylrhodamine methylester**	Fisher Scientific,Waltham, MA	T668

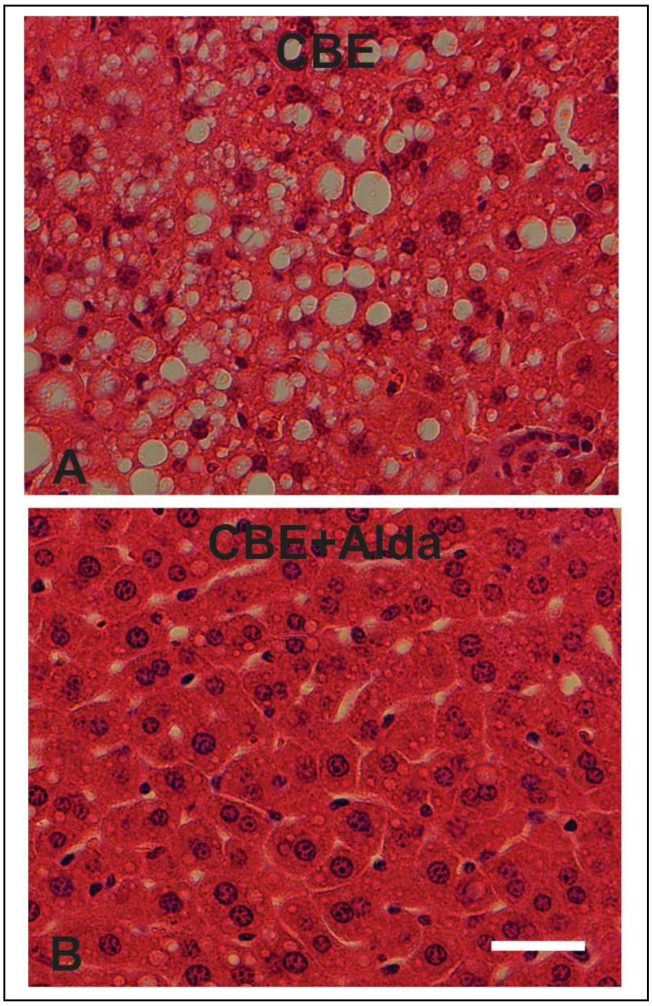

Figure S1. Attenuation by Alda-1 of chronic-binge ethanol-induced hepatic pathological changes in male mice. Male C57Bl/6 mice were fed an ethanol liquid diet and gavaged with one dose of ethanol (5 g/kg) on Day 16 of the protocol. Mice also received vehicle (**A**) or Alda-1 (**B**). Livers were harvested 9h after gavage. Shown are representative images of H&E-stained liver sections. Bar is 20μm.

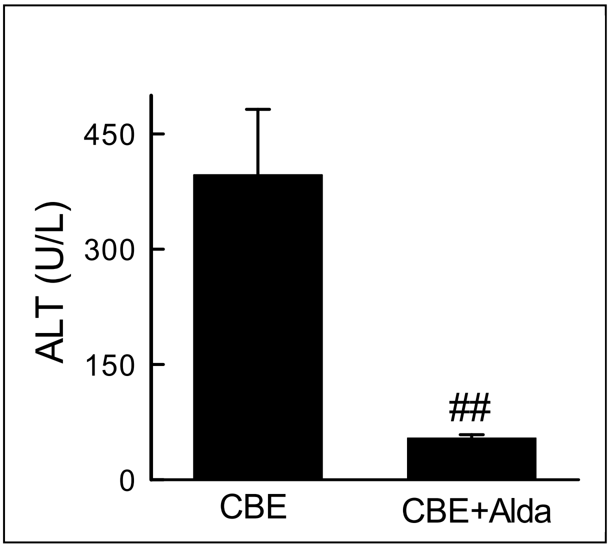

Figure S2. Liver injury after chronic-binge ethanol treatment in male mice: protection by Alda-1. Conditions as in Fig. S1. Serum was collected 9h after CBE. Serum ALT was detected using a kit. ##, $p<0.01$ vs CBE, determined by Student's t test. Values are means ± SEM (n = 4/per group).
